# Can untreated PKU patients escape from intellectual disability? A systematic review

**DOI:** 10.1186/s13023-018-0890-7

**Published:** 2018-08-29

**Authors:** Danique van Vliet, Annemiek M. J. van Wegberg, Kirsten Ahring, Miroslaw Bik-Multanowski, Nenad Blau, Fatma D. Bulut, Kari Casas, Bozena Didycz, Maja Djordjevic, Antonio Federico, François Feillet, Maria Gizewska, Gwendolyn Gramer, Jozef L. Hertecant, Carla E. M. Hollak, Jens V. Jørgensen, Daniela Karall, Yuval Landau, Vincenzo Leuzzi, Per Mathisen, Kathryn Moseley, Neslihan Ö. Mungan, Francesca Nardecchia, Katrin Õunap, Kimberly K. Powell, Radha Ramachandran, Frank Rutsch, Aria Setoodeh, Maja Stojiljkovic, Fritz K. Trefz, Natalia Usurelu, Callum Wilson, Clara D. van Karnebeek, William B. Hanley, Francjan J. van Spronsen

**Affiliations:** 1 University of Groningen, University Medical Center Groningen, Beatrix Children’s Hospital, 9700 RB Groningen, The Netherlands; 2 Department of Gastroenterology, Radboud University Medical Center, Nijmegen, The Netherlands; 3 Department of PKU, Kennedy Center, Copenhagen University Hospital, Glostrup, Denmark; 4 University Children’s Hospital, Jagiellonian University, Krakow, Poland; 5 Dietmar-Hopp Metabolic Center, University Children’s Hospital, Heidelberg, Germany; 6 Department of Pediatrics, Cukurova University Faculty of Medicine, Adana, Turkey; 7 Medical Genetics, Sanford Health, Fargo, ND USA; 8 Mother and Child Health Care Institute of Serbia Dr Vukan Cupic, School of Medicine, University of Belgrade, Belgrade, Serbia; 9 Department of Medical, Surgical and Neurological Sciences, Medical School, University of Siena, Policlinico Santa Maria Alle Scotte, Siena, Italy; 10 Department of Pediatrics, Hôpital d’Enfants Brabois, CHU Nancy, Vandoeuvre les Nancy, France; 11 Department of Pediatrics, Endocrinology, Diabetology, Metabolic Diseases and Cardiology of the Developmental Age, Pomeranian Medical University, Szczecin, Poland; 12 Department of General Pediatrics, Division of Neuropediatrics and Metabolic Medicine, Center for Pediatric and Adolescent Medicine, University Hospital Heidelberg, Heidelberg, Germany; 13 Department of Pediatrics, Tawam Hospital, Al-Ain, United Arab Emirates; 14 Department of Internal Medicine, Division of Endocrinology and Metabolism, Academic Medical Center, Amsterdam, Netherlands; 15 Department of Pediatrics, Oslo University Hospital, Oslo, Norway; 16 Clinic for Pediatrics, Inherited Metabolic Disorders, Medical University of Innsbruck, Innsbruck, Austria; 17 Metabolic Disease Unit, Sheba Medical Center, Edmond and Lily Safra Children’s Hospital, Tel Aviv, Israel; 18 Department of Pediatrics, Child Neurology and Psychiatry, Sapienza University of Rome, Rome, Italy; 19 Department of Internal Medicine, Oslo University Hospital, Oslo, Norway; 20 Genetics Division, Department of Pediatrics, Keck School of Medicine, University of Southern California, California, Los Angeles USA; 21 Department of Clinical Genetics, United Laboratories, Tartu University Hospital and Institute of Clinical Medicine, University of Tartu, Tartu, Estonia; 22 Department of Genetics and Metabolism, Chapel Hill hospital, University of North Carolina, Chapel Hill, USA; 23 Department of Chemical Pathology and Metabolic Medicine, Guys and St Thomas’ Hospitals NHS foundation trust, London, UK; 24 Department of General Pediatrics, Muenster University Children’s Hospital, Muenster, Germany; 25 Department of Pediatrics, Tehran University of Medical Sciences, Tehran, Iran; 26 Institute of Molecular Genetics and Genetic Engineering, University of Belgrade, Belgrade, Serbia; 27 Institute of Mother and Child, Centre of Reproductive Health and Medical Genetics, Chisinau, Moldova; 28 Newborn Metabolic Screening Unit, LabPlus, Auckland City Hospital, Auckland, New Zealand; 29 Departments of Pediatrics and Clinical Genetics, Academic Medical Centre, Emma Children’s Hospital, Amsterdam, The Netherlands; 30 Department of Pediatrics, Centre for Molecular Medicine and Therapeutics, University of British Columbia, Vancouver, Canada; 31 Clinical and Biochemical Genetics, Department of Pediatrics, The Hospital for Sick Children and the University of Toronto, Toronto, Canada

**Keywords:** Phenylketonuria, Phenylalanine, Late-diagnosed, Untreated, Brain vulnerability, Intellectual disability

## Abstract

**Background:**

Phenylketonuria (PKU) is often considered as the classical example of a genetic disorder in which severe symptoms can nowadays successfully be prevented by early diagnosis and treatment. In contrast, untreated or late-treated PKU is known to result in severe intellectual disability, seizures, and behavioral disturbances. Rarely, however, untreated or late-diagnosed PKU patients with high plasma phenylalanine concentrations have been reported to escape from intellectual disability. The present study aimed to review published cases of such PKU patients.

**Methods:**

To this purpose, we conducted a literature search in PubMed and EMBASE up to 8th of September 2017 to identify cases with 1) PKU diagnosis and start of treatment after 7 years of age; 2) untreated plasma phenylalanine concentrations ≥1200 μmol/l; and 3) IQ ≥80. Literature search, checking reference lists, selection of articles, and extraction of data were performed by two independent researchers.

**Results:**

In total, we identified 59 published cases of patients with late-diagnosed PKU and unexpected favorable outcome who met the inclusion criteria. Although all investigated patients had intellectual functioning within the normal range, at least 19 showed other neurological, psychological, and/or behavioral symptoms.

**Conclusions:**

Based on the present findings, the classical symptomatology of untreated or late-treated PKU may need to be rewritten, not only in the sense that intellectual dysfunction is not obligatory, but also in the sense that intellectual functioning does not (re)present the full picture of brain damage due to high plasma phenylalanine concentrations. Further identification of such patients and additional analyses are necessary to better understand these differences between PKU patients.

**Electronic supplementary material:**

The online version of this article (10.1186/s13023-018-0890-7) contains supplementary material, which is available to authorized users.

## Background

Phenylketonuria (PKU; OMIM 261600) is an inborn error of metabolism, characterized by impaired activity of the hepatic enzyme phenylalanine hydroxylase (PAH; EC 1.14.16.1) that normally converts phenylalanine (Phe) to tyrosine. Since the discovery of increased plasma Phe concentrations (hyperphenylalaninemia) as the underlying cause of intellectual disability (ID), (often intractable) seizures, and severe behavioral disturbances by Følling in the 1930s [1], two developments have strongly influenced the course of the disease. In the 1950s, it was first shown by Bickel that early institution of a Phe-restricted diet could prevent severe neurocognitive dysfunction [2]. In the 1960s, a diagnostic test was developed by Guthrie that enabled mass screening for hyperphenylalaninemia [3]. As a consequence, PKU became a model for other inborn errors of metabolism, as it was the first disorder in which severe neurocognitive dysfunction could be prevented by early initiation of treatment, the first disorder in which a ‘simple’ diet rather than a drug was the intervention, and the first disease ever for which early diagnosis was possible due to population based neonatal screening [4].

Today, 100 years after the birth of Guthrie, most countries in the developed world have adopted population-based neonatal screening for PKU [5], and each infant with confirmed PKU is immediately put on a Phe-restricted diet to reduce plasma Phe concentrations to levels within the target range. This combination of early diagnosis and initiation of treatment has resulted in normal IQ for most PKU patients [6]. To aim at optimal neurocognitive and psychosocial outcome of PKU patients, the recommended upper target plasma Phe levels in both the first European guidelines and USA consensus statement are based on the assumption that the correlation between plasma Phe concentrations and neurocognitive/psychosocial outcome is the same for all PKU patients, which may not be true. At least some patients still show mild neurocognitive and psychosocial impairments, even when plasma Phe concentrations are only mildly elevated (600–1200 μmol/l) [7–9]. On the other hand, rarely, untreated or late-diagnosed PKU patients with very high plasma Phe concentrations (> 1200 μmol/l) have been reported to escape from ID [10, 11].

To investigate what these patients can teach us about the inter-individual differences in brain vulnerability to high plasma Phe between PKU patients, this study aimed to review published cases of late-diagnosed PKU patients without ID despite high plasma Phe concentrations.

## Methods

### Search strategy

We initially conducted a literature search on PubMed and EMBASE without date limits up to 10th of August, 2016. In both PubMed and Embase, a search including the following keywords (Mesh) and free text terms in titles and abstracts (tiab) was entered: (“Phenylketonurias”[Mesh] OR phenylketonuria*[tiab] OR pku [tiab] OR Oligophrenia Phenylpyruvica [tiab]) AND (atypical*[tiab] OR late diagnos*[tiab] OR late treat*[tiab] OR late detect*[tiab] OR adult-onset [tiab] OR untreat*[tiab] OR normal intelligen*[tiab] OR above average intelligen*[tiab] OR normal intellect*[tiab] OR normal IQ [tiab] OR borderline intelligen*[tiab] OR undiagnos*[tiab] OR unrecogni*[tiab] OR mild phenylketonuria [tiab] OR mild pku [tiab] OR mild hyperphenylalaninemia [tiab]). This search was updated on 3rd of February and 8th of September 2017.

### Study and case selection

**Fig. 1:**
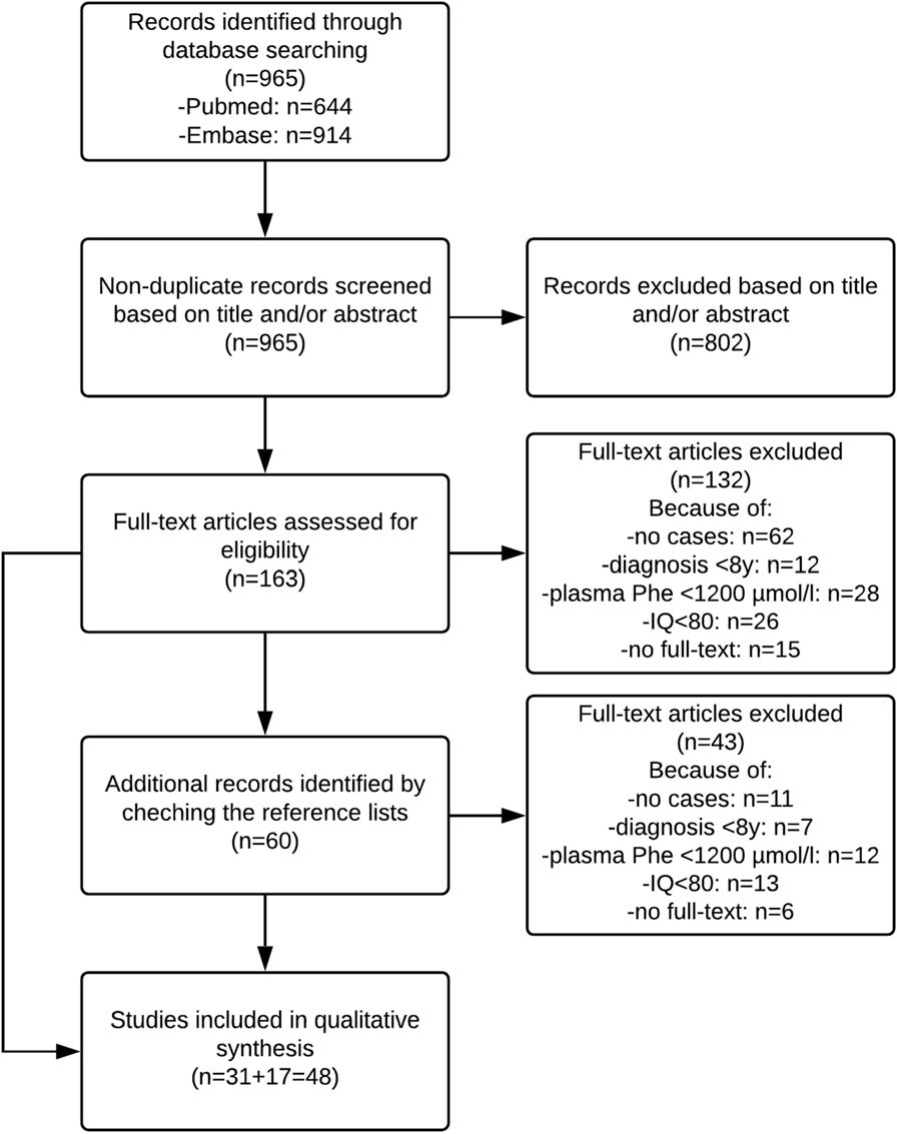
Outline of the reviewing process of the systematic literature search as performed by DvV and AMJvW

First, titles and/or abstracts of all identified non-duplicate references were screened to select eligible studies. Eligibility criteria included: 1) PKU patients being late-diagnosed/−treated, and 2) information on IQ and plasma Phe concentrations being available. Then, full-text articles of the selected references were retrieved and read independently by two authors (DvV, AMJvW) to assess whether the inclusion criteria were met. Studies were included if they described at least one case meeting the following criteria: 1) PKU diagnosis and/or start of treatment after 7 years of age (based on the definition of untreated PKU as referring to patients who are untreated at age 7 years or older [12]); 2) untreated plasma Phe concentrations ≥1200 μmol/l; and 3) IQ ≥80 (based on most previously used IQ scoring systems that defined a normal intelligence as an IQ ≥80). This combination of inclusion criteria was aimed to identify those PKU patients representing the one end of the phenotypic spectrum with regard to neurocognitive outcome in relation to plasma Phe levels in untreated PKU patients. Studies not describing detailed information on an individual PKU patient were excluded. The reference lists of all full-read articles were reviewed to identify additional eligible studies. Selection of eligible articles, selection of articles to be included, and extraction of data from selected articles was performed independently by DvV and AMJvW. Any inconsistencies were solved by discussion among DvV, AMJvW, and FJvS. Results of the reviewing process are outlined in Fig. 1. Physicians/treating centers were contacted for possible further information about previously described cases that were included in our study.

## Results

In total, we identified 59 reported cases of late-diagnosed (>7y) PKU patients without ID (as defined by an IQ ≥80), despite untreated plasma Phe concentrations of ≥1200 μmol/l (Additional file 1). Of all 59 reported cases (Table 1), most patients had been diagnosed because of a sibling with PKU, or because they had given birth to children with PKU or children suffering from the maternal PKU syndrome. In addition, ten cases were identified by screening programs at adulthood. Most of these screening programs were performed in women before or during pregnancy to identify those being at risk of bearing children suffering from the maternal PKU syndrome. In eight cases, the reason for diagnosis was different or not reported (cases #32 and #44). Case #9 was diagnosed because of high neonatal plasma Phe concentrations in her child without any underlying enzymatic defect in the offspring, and case #18 was diagnosed by a survey performed in the hospital [13]. Other cases were diagnosed because of cerebral symptoms. Case #2 presented at childhood with hyperactivity [14, 15]. Cases #41 and #42 presented at adolescence with neurological symptoms (tremor and amaurosis fugax) but intact intellectual functioning [16], and case #52 presented only at the age of 57 years with progressive spastic paraparesis and dementia for four years [17]. Remarkably, case #52 also had a late-diagnosed PKU sibling, but had never been investigated for PKU. Of the 11 reported patients diagnosed with PKU between 1-7y, three (27%) were diagnosed following the identification of PKU in a sibling or relative, two (cases #62 and #65) because of a positive urine ferric chloride test on routine examination [18, 19], case #60 because of the smell of phenylacetic acid [20], and two patients because of developmental delay (case #63 and #66) [15, 21].

**Table 1:** Characteristics of late-diagnosed (> 7 years) PKU patients who have escaped from intellectual disability despite high plasma Phe concentrations

Patient characteristics	Frequency (percentage)
Gender:	
-male	12 (20%)
-female	45 (76%)
-not reported	2 (3%)
Reason diagnosis:	
-PKU sibling	19 (32%)
-PKU offspring	8 (16%)
-affected offspring	19 (32%)
-screening	10 (17%)
-PKU relative	1 (2%)
-other	6 (10%)
-not reported	2 (3%)
Genetic confirmation of PAH deficiency	
-yes	14 (24%)
-not reported	45 (76%)
Intellectual Quotient (IQ):	
80–90	17 (29%)
90–100	16 (27%)
100–120	12 (20%)
≥ 120	2 (3%)
“normal”	12 (20%)
Neurological outcome:	
-abnormal	17 (68%)
-no abnormalities (reported)	8 (32%)
Psychological/psychiatric/social outcome:	
-abnormal	10 (48%)
-no abnormalities (reported)	11 (52%)

Regarding the neurological outcome, of all 59 cases, no (0%) seizures were described, but 4/10 were reported to have an abnormal EEG. In addition, 12 cases (20%) showed other neurological symptoms, primarily including abnormal reflexes, movement disorders, and motor difficulties. While, according to the inclusion criteria, intellectual outcome was within the normal range for all patients, ten patients (17%) had one or more problems in neuropsychological or social functioning.

For 6 cases, additional neuroimaging and/or biochemical information was provided. Cases #43 and #44 were described to show only mild cerebral MRI abnormalities and brain Phe levels as determined by magnetic resonance spectroscopy (MRS) < 0.02 mmol/l, despite plasma Phe concentrations > 1200 μmol/l [22, 23]. Also, no cerebral MRI abnormalities were observed in case #52 who presented in adulthood with progressive spastic paraparesis, dementia for four years, and high plasma Phe concentrations [17], while cases #41 and #42 showed MRI involvement scores that were comparable with other late-diagnosed PKU patients [16]. Case #2, diagnosed at 9 years of age because of hyperactivity and poor motor performance but normal IQ, was the one patient for whom CSF analyses were reported, showing an elevated Phe concentration of 456 μmol/l (at plasma Phe of 1140–1500 μmol/l) [14].

Besides outcomes with respect to the central nervous system, for some included PKU patients, physical characteristics have been reported as well (data not shown). Many of these PKU patients showed the typical physical characteristics of untreated PKU: fair skin, blond hair, blue eyes, and sometimes also eczema.

## Discussion

This study describes cases of PKU patients without ID despite late diagnosis with high plasma Phe concentrations, representing one of the old, but still unresolved, questions in PKU. Most notable is the fact that we identified so many published cases with unexpected favorable outcome. The second important observation was that, although these patients had intellectual functioning within the normal range (IQ ≥80), many showed other (mild) cerebral PKU symptoms. The third remarkable finding was that, in some of the PKU patients, neurological symptoms only started at adult age, although this can still also be due to another disease rather than PKU.

Classical symptomatology of untreated or late-treated classical PKU consists of severe to global developmental delay, seizures, psychiatric disorders, and profound ID, with IQ declining to 40 or lower at one year of age [24]. Early natural history and cohort studies, however, also showed that this severe clinical picture does not apply to all PKU patients, postulating that approximately 1–2% of the total PKU population would somehow have escaped from ID [10, 25]. However, based on the number of identified PKU patients born before the introduction of neonatal screening with severe ID that seems to be far less than would be expected from the current prevalence of classical PKU patients found at neonatal screening [26], the incidence of such “unusual” PKU may be higher than previously thought and many “unusual” PKU patients seem to have remained unidentified. This is further substantiated by the number of classical PKU females with normal intelligence who have been identified only because of their children showing the maternal PKU syndrome, resulting in 45 females and only 12 males being included in Table 1, suggesting that especially “unusual” male PKU patients have remained unidentified. More recent calculations based on screening programs for hyperphenylalaninemia in pregnant women estimate that the percentage of “unusual” PKU patients may be closer to 10% [27].

**Fig. 2:**
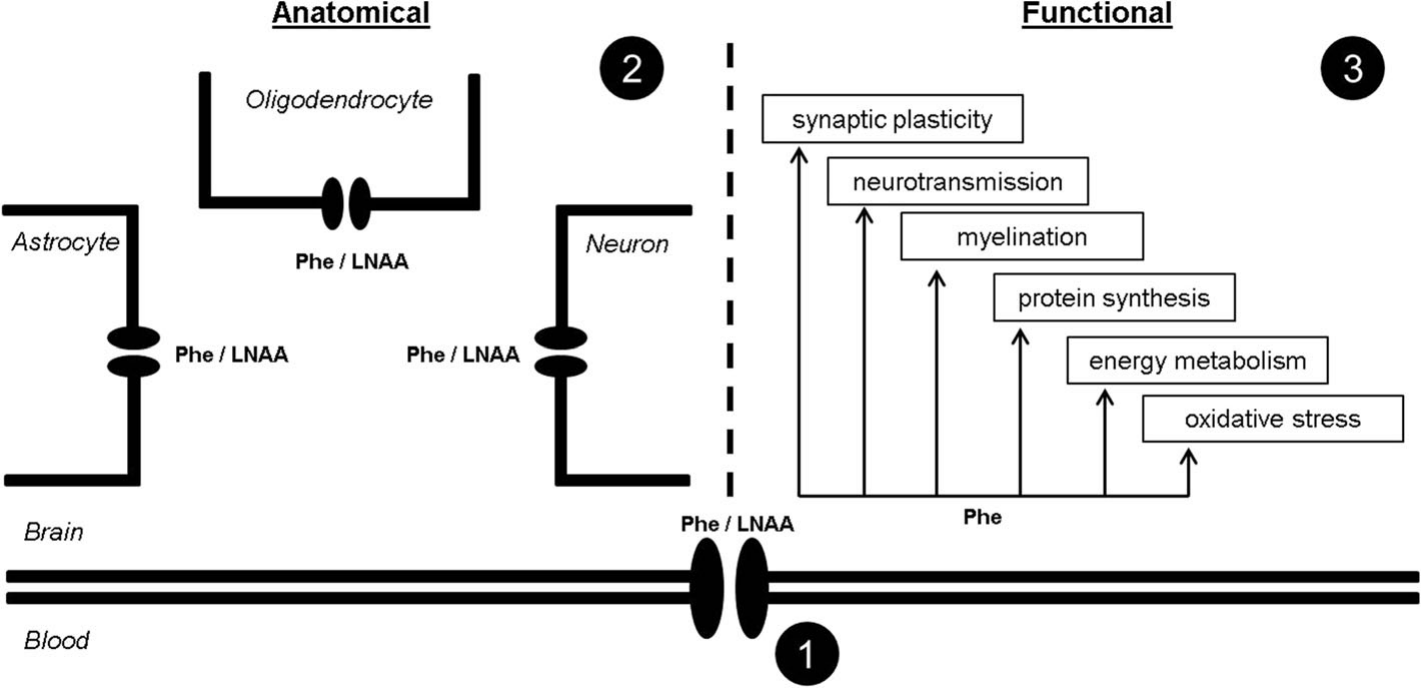
Schematic picture outlining the hypotheses regarding the possible mechanism (s) underlying the inter-individual differences in brain vulnerability to high plasma Phe concentrations between PKU patients including: 1) a difference in the transport of Phe and other large neutral amino acids across the blood-brain barrier, 2) a difference in the transport of Phe and other large neutral amino acids across membranes of different cell types within the brain, and 3) a difference in the vulnerability of one or more of the intracerebral processes to high brain Phe concentrations

Besides the question how many PKU patients should be classified as “unusual” (and might currently be overtreated), the mechanisms underlying the lack of ID without early diagnosis and treatment remain unresolved. It has been hypothesized that these patients may have some protecting mechanism, located at the blood-brain barrier or within the brain itself, that is involved in Phe transport or metabolism, or in the cerebral responses to high brain Phe concentrations [22, 23] (Fig. 2). At the level of the blood-brain barrier, LAT1 is considered to be the predominant transporter for Phe and other large neutral amino acids, and as such has been hypothesized to play a role in the inter-individual differences in brain vulnerability to high plasma Phe concentrations between PKU patients [28]. However, the transport of Phe and other large neutral amino acids across membranes of different cell types within the brain is less well understood. In support of a possible protecting mechanism located either at the blood-brain barrier or within the brain itself, many “unusual” patients in the current review, besides high plasma Phe concentrations, showed the physical PKU characteristics of fair skin, blond hair, and blue eyes. Interestingly, however, many of the here presented cases with normal intellectual functioning show some other cerebral (e.g. neurological or neuropsychological) PKU symptoms. Moreover, in contrast to the hypothesis of a possible variation in Phe transport from blood to brain in these patients, Phe concentrations in CSF of case #2 were correspondingly high with their plasma Phe concentrations [14].

## Conclusion

To conclude, the old “unusual” PKU cases as described in the present review give us more than the simple information that they indeed exist. These cases at least suggest that, even when ID is not seen, other neuropsychiatric symptoms may still exist, suggesting that the pathophysiology of brain dysfunction in PKU might relate to more than one mechanism. We, therefore, do not only need precise description of late-diagnosed PKU patients with unexpected favorable outcome despite high plasma Phe, but also need to further investigate these cases by modern techniques such as metabolomics and next generation sequencing to define the exact underlying mechanisms of PKU brain dysfunction. The fact that more and more PKU patients are now diagnosed and treated from birth further necessitates that we really find these patients right now.

## Additional file

Additional file 1: **Table S1.** Reported and previously unreported cases of late-diagnosed (> 7 years) PKU patients who have escaped from intellectual disability despite high plasma Phe concentrations. (DOCX 52 kb)

## Abbreviations

BBB: Blood-brain barrier
ID: Intellectual disability
LNAA: Large neutral amino acids
PAH: Phenylalanine hydroxylase
Phe: Phenylalanine
PKU: Phenylketonuria
