# Supplementary material for: Can untreated PKU patients escape from intellectual disability? A systematic review
Source: Orphanet J Rare Dis. 2018 Aug 29;13:149. doi: 10.1186/s13023-018-0890-7 (PMC6116368; doi:10.1186/s13023-018-0890-7)
Supplement: Supplementary file 1 — Table S1. Reported and previously unreported cases of late-diagnosed (> 7 years) PKU patients who have escaped from intellectual disability despite high plasma Phe concentrations. (DOCX 52 kb) [file 13023_2018_890_MOESM1_ESM.docx]

**Table S1. Reported and previously unreported cases of late-diagnosed (>7 years) PKU patients who have escaped from intellectual disability despite high plasma Phe concentrations**

| **Case** | **Age at diagnosis (M/F)** | **Reason diagnosis** | **Phe (µmol/l)** | **PAH deficiency** | **IQ** | **Neurological** | | **Psychological/psychiatric/social** | |
| --- | --- | --- | --- | --- | --- | --- | --- | --- | --- |
|  |  |  |  |  |  | **Normal findings** | **Abnormal findings** | **Normal findings** | **Abnormal findings** |
| 1[12] | 12y (M) | A | 1860 | NR | 85_1_ |  | stiff, clumsy motoric abilities, slightly prostrate posture |  |  |
| 2[13] | 9y,11m (M) | F, hyperacti-vity | 1140-1500 | NR | 107_2_-109_3_ | normal reflexes | EEG abnormal: paroxysmal and disorganized; but poorly organized motor function, which was not improved after institution of diet |  | hyperactive, anxious, extremely excited before any disruption of the routine, considerable perseveration, specific deficit in perceptual visual motor abilities, and poor impulse control |
| 3[14] | 9y (F) | A | 1020-1500 | NR | 95_3_ | normal milestones, neurological examination within normal limits, continued to show normal intellectual capacity despite no institution of treatment |  | good deal of anxiety and inhibition | lack of childhood spontaneity with related ambivalence in acting out her emotional impulses |
| 4[15] | 8y (M) | A | 1680 | NR | 91 |  | ataxia, very mild choreoathetoid-like movements which could be from irritable restlessness and poor coordination, slight difficulty in speech which was attributed to poor tongue coordination, some slowness in reading |  | nervousness |
| 5[16, 17] | 8y (M) | A | 1560 | NR | 89_2_ | NR | NR | NR | NR |
| 6[18, 19] | 36y (F) | B | 1236-1248 | NR | 102_4_ | NR | mild reading disability | NR | NR |
| 7[20] | 12y (M) | A | 1200-1500 | NR | 91-100 | NR | NR | NR | NR |
| 8[21] | 12y (F) | A | 1920 | NR | 106 | no abnormalities on neurological examination; EEG borderline normal |  | bright, quiet and confident |  |
| 9[22] | 26y (F) | F,  high Phe in child without enzymatic defect | 1200-1800 | NR | 96_4_ | NR | NR | NR | NR |
| 10[23] | 29y (F) | B | 1020-1320 | NR | ≥90_4_ | no neurological abnormalities on examination | EEG: mildly abnormal paroxysmal record; very brisk tendon reflexes | NR | NR |
| 11[24] | 34y (F) | C | 1320-1560 | NR | 97_4_ | NR | NR | NR | NR |
| 12[24] | adult (F) | C,E | 1560-1680 | NR | 92_2_ |  | did not begin talking as early as her brother | NR | NR |
| 13[25] | 32y (F) | B | 1302-1560 | NR | 88_2_ | no seizures | EEG low voltage, diffuse slowing, and paroxysmal generalized high voltage | no psychic disturbances |  |
| 14[26] | adult (F) | B,C | 1500-2580 | NR | 84-90_4_ | no abnormalities on neurological examination, EEG normal |  | very apt in nursing her three invalid children, coped well with family illnesses |  |
| 15[10] | 7y,4m (F) | A | 1560 | NR | 104_6_-98_2_ | NR |  | NR | NR |
| 16[27] | 8y (M) | A | 1080-1320 | NR | 93_3_ | EEG normal |  | NR | NR |
| 17[27] | 12y (F) | A | 960-1200 | NR | 91_3_ | EEG normal |  | NR | NR |
| 18[27] | 68y (M) | F,  survey in hospital | 1200-1500 | NR | >80 | developed normally until 2 years | when he suffered from meningitis, thereafter he required 6 months to recover normal motor function but was considered intellectually retarded |  | admitted to sheltered environment after having been convicted for which he was considered innocent |
| 19[28, 29] | 29y (F) | B | 1560-1860 | NR | * | NR | NR | NR | NR |
| 20[30, 31] | 28y (M) | A | 1370-1510 | NR | 120_3_-132_2_ | no seizures, no other neurological abnormalities | EEG mild temporal dysrhythmia | NR | NR |
| 21[32] | 11y (M) | A | 1200 | NR | 104_5_ | NR | NR |  | very tense and anxious and defends against this by clowning, pressure of talk, and a rather uninhibited call for reassurance |
| 22[17, 33] | 9y (F) | A | 1500 | NR | * | no abnormalities on neurological examination |  |  | nervy, fearful, and easily upset at 11 years, and a little tense at 17 years |
| 23[17] | >12y (M) | A | 1260 | NR | * |  | microcephaly, slight fine tremor both hands, bilateral patellar clonus |  | immature, considerable abnormality with respect to his adjustment, neuropsychological subtest scores indicate emotional disturbance rather than the effect of brain damage, some difficulties with non-verbal abilities |
| 24[34] | adult (M) | D | 1332 | NR | 100 | NR | NR | NR | NR |
| 25[35] | 19y (F) | D | 1939 | NR | 122 | NR | NR | NR | NR |
| 26[35] | adolescence (F) | A | 1735 | NR | * | NR | NR | NR | NR |
| 27[35] | 24y (F) | D | 2655 | NR | 86 | NR | NR | NR | NR |
| 28[36] | 22y (F) | D |  | NR | 93_4_ | EEG within normal limits | hyperactive tendon reflexes, downgoing plantar reflexes | NR | NR |
| 29[37] | 28y (F) | B,C | 1200 | NR | 89_6_ | NR | NR | Inconspicuous |  |
| 30[38] | 27y (F) | D | 1340 | NR | 92_4_ | NR | NR | NR | NR |
| 31[11] | 21y (F) | A | 1500-1700 | NR | 82_4_ | normal muscle coordination (won open art competition) |  | NR | NR |
| 32[39] | adult (F) | NR | 1520 | NR | 97 | NR | NR | NR | NR |
| 33[40] | 35y (F) | C | 1374 | NR | 107_7_ | NR | NR | held responsible job |  |
| 34[40] | 64y (F) | C | 1588 | NR | 85_10_ | NR | NR | held responsible job |  |
| 35[41] | adult (F) | C | 2100 | NR | * | NR | NR | NR | NR |
| 36[42] | 23y (F) | A,C | 1346 | Yes | * | NR | NR | socially well adjusted, very little suggested PKU in her behavior |  |
| 37[42] | 24y (F) | C | 1265 | Yes | * | NR | NR | socially well adjusted, very little suggested PKU in her behavior |  |
| 38[43] | 22y (F) | C | 1423-1643 | NR | * | NR | NR | no social and behavioural problems |  |
| 39[44] | adult (F) | B,C | 1200 | Yes | 100 | NR | NR | socially well adjusted, works as school teacher |  |
| 40[45] | adult (F) | C | >1200 | Yes | * | NR | NR | NR | NR |
| 41[46] | 11y (M) | F,  tremor | *1740* | NR | *106*_5_ | *no history of seizures. EEG: no abnormalities No abnormalities on cerebral CT.* | *from 9y: distal upper limb tremor (shaking hands), dysgraphy, problems in fine motor skills, worsening school performances. At 17y: dysmetabolic leukoencephalopathy with anterior and posterior periventricular white matter abnormalities on MRI* | *high school graduate* | *irritability* |
| 42[46] | 18.4y (F) | F,  amaurosis fugax | 1500-1550 | NR | 85 |  | squint and amaurosis of the left eye, white matter involvement score 8, learning difficulties | NR | NR |
| 43[47-49] | 24y (F) | C | 1210-1800 | Yes | 100_4_ |  | only mild changes on MRI confined to parieto-occipital region | NR | NR |
| 44[47, 48] | NR (F) | NR | 1200 | Yes | 105_4_ |  | only mild changes on MRI confined to parieto-occipital region | NR | NR |
| 45[50] | 32y (F) | A,C | 1745 | Yes | 92 | NR | NR | NR | NR |
| 46[50] | 21y (F) | C | 1273 | NR | * | NR | NR | NR | NR |
| 47[50] | 25y (F) | C | 1739 | Yes | 100 | NR | NR | NR | NR |
| 48[50] | 20y (F) | D | 1376 | NR | 105 | NR | NR | NR | NR |
| 49[51] | adult (F) | C | 1380 | NR | 90_4_ | no PKU phenotype |  | no PKU phenotype |  |
| 50[52] | NR | A | 1274 | Yes | >85_6_ | NR | NR | NR | NR |
| 51[52] | NR | A | 1470 | Yes | >85_6_ | NR | NR | NR | NR |
| 52[53] | 57y (F) | F,  progres-sive spastic parapare-sis and dementia | 1862-2153 | Yes | 108 | normal neurodevelopmental history, normal visual and somatosensory evoked potentials, on MRI no white matter abnormalities | since some years progressive spastic paraparesis and dementia, brisk reflexes, ankle clonus, upgoing left toe |  | never employment/ driving licence/ dated/married, now flattened affect, marked psychomotor slowing |
| 53[54] | adult (F) | D | >1200 | NR | >80 | NR | NR | NR | NR |
| 54[54] | adult (F) | D | >1200 | NR | >80 | NR | NR | NR | NR |
| 55[54] | adult (F) | D | >1200 | NR | >80 | NR | NR | NR | NR |
| 56[54] | adult (F) | D | >1200 | NR | >80 | NR | NR | NR | NR |
| 57[55] | adult (F) | C | 731-1232 | Yes | * | NR | NR | NR | NR |
| 58[56] | adult (F) | C | 1560 | Yes | * | NR | NR | NR | NR |
| 59[57] | adult (F) | B | 850-1300 | Yes | *87*_8_ |  | tremor |  | *some problems in executive functioning: difficulty planning, reduced working memory, difficulty dividing attention, and* attention deficit, hyperactivity |

NR: not reported; A: PKU sibling; B: PKU offspring; C: affected offspring; D: screening; E: PKU relative; F: other

IQ assessment by: _1_ Binet-Simon-Kramer; _2_ Stanford-Binet; _3_ WISC; _4_ WAIS; _5_ Terman-Merrill; _6_ Hawik-Test; _7_ Raven’s Standard Progressive Matrices; _8_ Merill-Palmer Scale; _9_ Witti; _10_ estimated; *reported to have a “normal intelligence”

Additional Information provided by the physicians/treating centers of previously described cases is stated in italics.
